# Traces of early developmental bias in the adult brain

**DOI:** 10.1038/s41598-023-38371-8

**Published:** 2023-07-25

**Authors:** Gad Serero, Maria Lev, Dov Sagi, Uri Polat

**Affiliations:** 1 School of Optometry and Vision Science, Mina and Everard Goodman, Faculty of Life Sciences, Bar-Ilan University, Ramat Gan, Israel; 2 The Weizmann Institute of Science, Rehovot, Israel

**Keywords:** Neuroscience, Sensory processing

## Abstract

During the first 2 years of life, there is a high prevalence of optical distortions in the human eye, causing vertical blur on the retina (astigmatism), which is naturally resolved by the age of 5; thus, it is not treated. Here we determined the possible long-term effects on visual grouping resulting from optical distortions during the development of visual perception. Our results show a clear directional bias in shape perception for optically corrected astigmatic adults, compared with non-astigmatic ones, with remarkably slow decision times. These effects can be explained by a mismatch between the developmental timescales of different components in the visual system.

## Introduction

During the early period of visual development, distorted visual inputs to the eye’s retina are considered normal and tend to disappear later. Most neonates and infants are farsighted (hyperopic)^1^, with a high prevalence of significant astigmatism (> 1 Diopters)^2–8^, which appears to be, in most individuals, of corneal origin^7,9,10^ (estimated 17% to 63% of the population^5,11^). Much of the astigmatism in infancy is resolved during the first 2–5 years of life^3,5,12,13^, depending on its severity^14^; thus, optical correction is not practiced^15^ under a certain level. After the age of 6, due to changes in eye refraction toward myopia and to increased lid pressure on the cornea^16^, astigmatism is mostly reduced to < 1 Diopter^13^. Most early astigmatism is characterized by vertical spatial distortion^5^, causing vertical blur (steep horizontal corneal meridian), so that a circular spot is projected as vertically elongated on the retina during early life. Maturation of neural connectivity is experience dependent; thus, adaptation to uncorrected visual input during development may cause long-term connectivity changes in the visual system, possibly persisting after the delayed maturation of the optics when the input bias is largely removed. Indeed, anisotropic contrast sensitivity in optically corrected individuals with high astigmatism was reported, suggesting that individual differences in contrast sensitivity may be traced to early development^17^. Here we aimed to determine the effects of anisotropic contrast sensitivity on shape perception at adulthood resulting from this mismatch in development between different components of the visual system.

**Figure 1:**
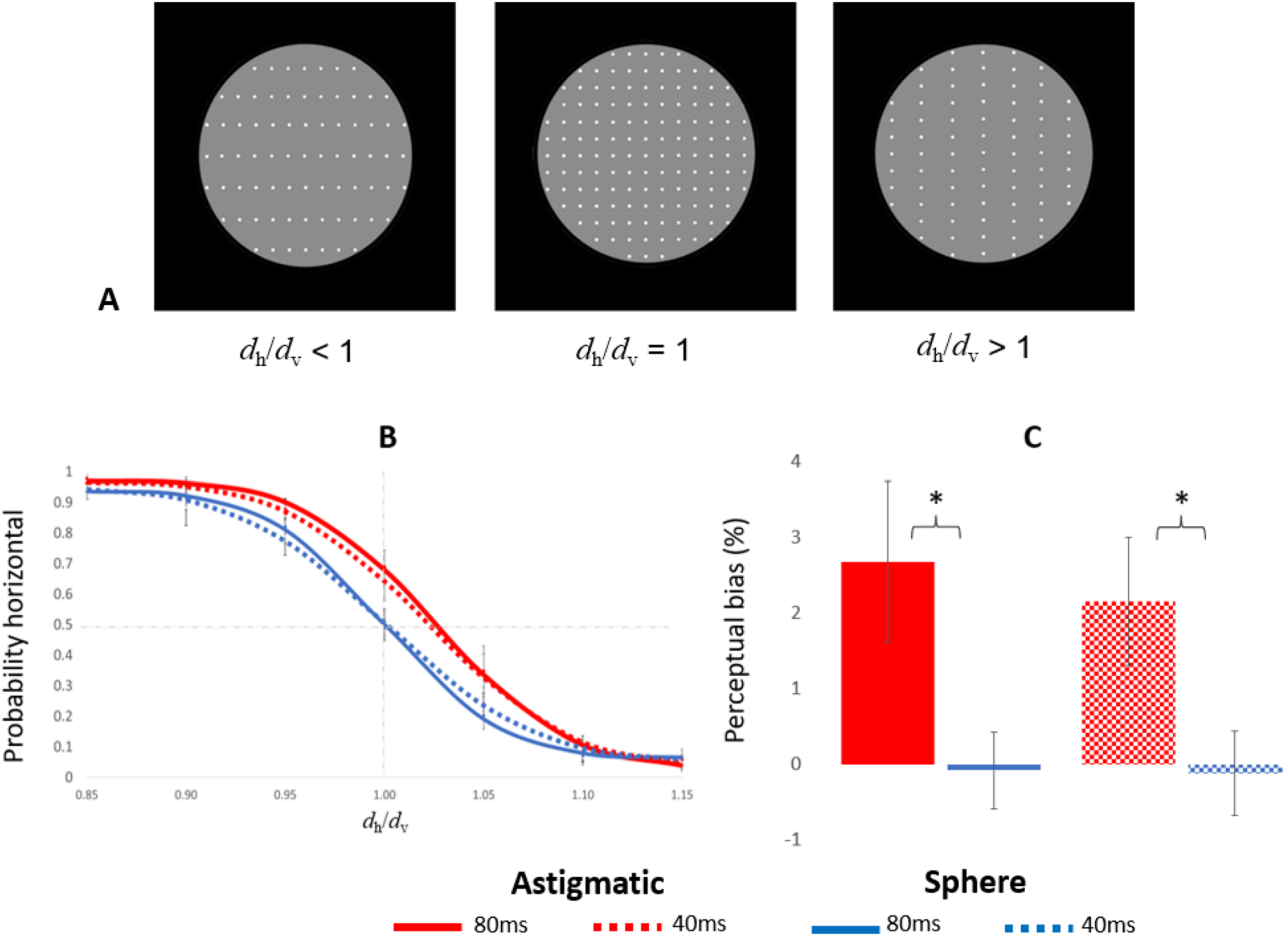
(**A**) Proximity grouping. Participants reported that they perceived the orientation of the dot patterns as horizontal or vertical (2-Alternative Forced Choice, 2AFC). Different inter-dot spacing ratios (5% steps) were presented to measure the proximity grouping performance. *d*_*h*_ represents the horizontal spacing and d_*v*_ the vertical spacing. The displays show examples when *d*_*h*_ < *d*_*v*_ perceived as rows, *d*_*h*_ = *d*_*v,*_ producing an ambiguous grouping, and *d*_*h*_ > *d*_*v*_ perceived as columns. (**B**) Psychometric curves depicting the probability of reported horizontal organization as a function of the spacing ratio, for spherical (N = 5, blue) and astigmatic (N = 5, red) groups, for stimulus durations of 40 and 80 ms. (**C**) Perceptual bias, measured as the *d*_h_/*d*_v_ deviation from 1, where performance is at chance level. The results show a significant bias, duration independent, for the astigmatic group but none for the spherical group. Error bars represent 1SEM.

Optically corrected astigmatic (0.75–1.5 D) and non-astigmatic (spherical) adults were tested with a perceptual grouping task^18^; they reported the perceived orientation of an array of dots (Fig. 1A). Optical correction was verified using a standard methodology (contrast sensitivity, artificial pupil, over refraction). Perception in this task is known to depend on the ratio between the horizontal and vertical spacing, *d*_h_/*d*_v_, with *d*_h_/*d*_v_ < 1 leading to perceived horizontal organization, *d*_h_/*d*_v_ = 1 showing no definite orientation, and *d*_h_/*d*_v_ > 1 leading to perceived vertical organization. The presence of vertical blur in the image is known to bias perception toward vertical organization^15^. Research^5^ suggests that children without astigmatism in infancy probably will not acquire it at a later age. Thus, adults with astigmatism are most likely to develop astigmatism during infancy. Therefore, we hypothesized that our astigmatic participants had the same or higher levels of astigmatism during their infancy, resulting in a more extensive and longer exposure to vertical blur. Thus, if visual plasticity decays before the eyes’ optics mature, a bias in orientation perception is expected.

## Results

Psychometric curves were generated for each participant. The probability of horizontal judgment (P_horizontal_) was calculated for each participant, for each *d*_h_/*d*_v_ condition, averaged across participants, and plotted in Fig. 1B. There was no significant difference between monocular and binocular conditions; thus, the psychometric curves represent the averages of these conditions.

Figure 1B presents psychometric curves obtained for the astigmatic and control groups (non-astigmatic) for stimulus durations of 80 and 40 ms. The control group (blue curve) shows the expected behaviour, with the probability of horizontal reports decreasing for increasing *d*_h_/*d*_v_, showing chance performance (P_*horizontal*_ = 0.5) at *d*_h_/*d*_v_ = 1, thus unbiased. However, the astigmatic group (red curve) shows chance performance at *d*_h_/*d*_v_ > 1, indicating a significant bias. Biases were computed for each participant as the deviation in *d*_h_/*d*_v_ from unity that yields chance behavior, using standard modeling methods (see the Methods section), with the estimated group biases depicted in Fig. 1C. Our results show that the astigmatic group have a significant perceptual bias (p < 0.01; one sample t-test, Cohen’s *d* = 1.85), whereas the spherical group did not (p = 0.21; one sample t-test). Comparing to spherical group, the bias is significantly higher for astigmatic participants (p < 0.01, Cohen’s *d* = 1.49). Moreover, the analysis by group and stimuli duration, shows a significant perceptual bias for the astigmatic group (respectively for 80 and 40 ms, Mean ± SE; 2.7% ± 1.07%, p = 0.016, Cohen’s *d* = 1.51 and 2.2% ± 0.85%, p = 0.02, Cohen’s *d* = 1.31), whereas the spherical group showed no significant bias with both stimulus durations (Mean ± SE; − 0.08% ± 0.51%, − 0.12% ± 0.56%). There was no significant effect of stimulus duration on the bias (Two-way ANOVA, F (1,16) = 0.15, p = 0.69); however, the psychometric curves are steeper in the 80 ms condition, showing a significant sensitivity difference compared to 40 ms (Pairwise t-test p = 0.03, Cohen’s *d* = 0.43). But no significant sensitivity difference has been observed between the study groups (Two-way ANOVA, F(1,16) = 1.33, p = 0.26).

### Reaction time

**Figure 2:**
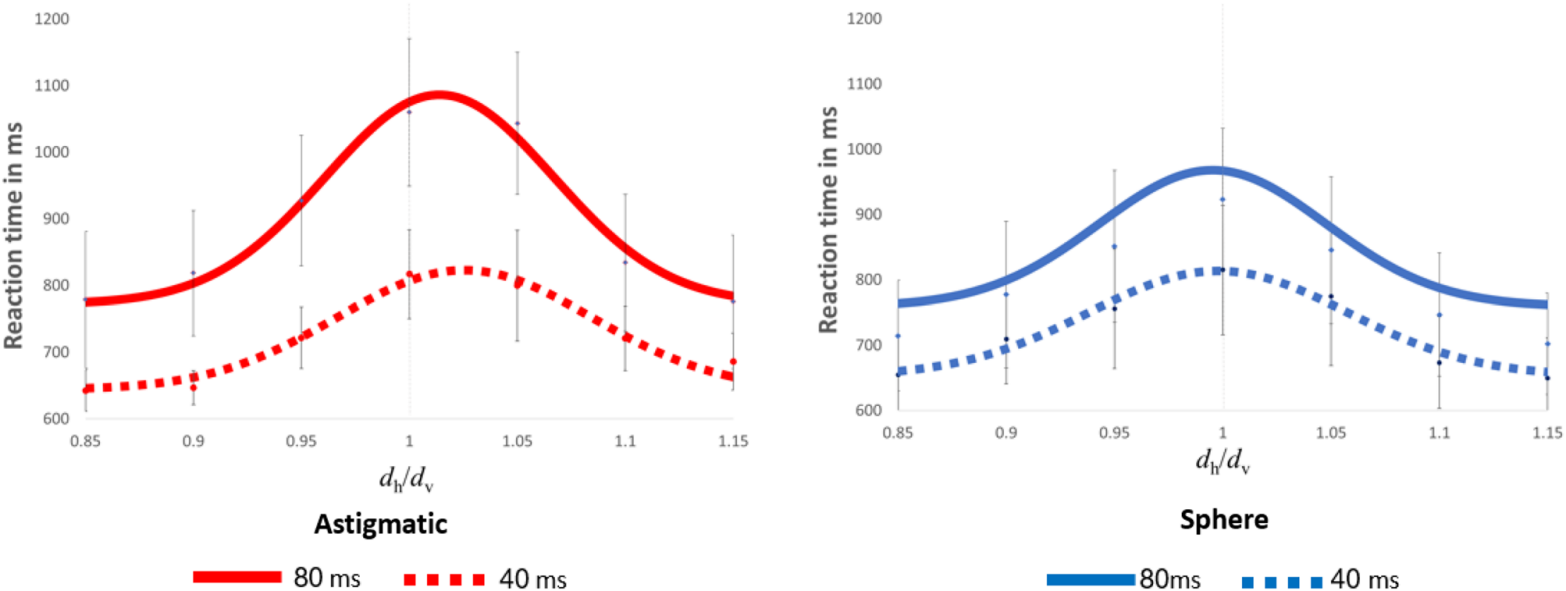
Median reaction times (RT), plotted as a function of the relative spacing, *d*h/*d*v. RT increases with stimulus ambiguity. Note the slower RT with the longer duration stimuli, enhanced in the astigmatic group. Error bars represent 1 SEM.

Figure 2 shows the median reaction time for the astigmatic and spherical (control) groups as a function of the spacing ratio between the dots. As expected, the reaction time slows down when a decision is difficult to make, i.e., when the stimulus interpretation is ambiguous. For the spherical group, the slower reaction times are around *d*_h_/*d*_v_ = 1, whereas for the astigmatic group, the slower reaction times are at *d*_h_/*d*_v_ > 1, indicating a perceptual bias. These results clearly show that both groups were faster when the stimuli were presented for 40 ms, compared with 80 ms (Two-way ANOVA, F(3,112) = 7.4, p < 0.01, Cohen’s *d* = 0.67). An analysis by group shows for the hard tasks (*d*_h_/*d*_v_ = 1) that the astigmatic group RT at 80 ms (1060 ms ± 110) is slower than at 40 ms (Mean ± SE; 817 ms ± 74; p < 0.01, Cohen’s *d* = 1.28), and for the spherical group at 80 ms (923 ± 109), it was slower than at 40 ms (815 ± 98; p = 0.03, Cohen’s *d* = 0.51).

**Figure 3:**
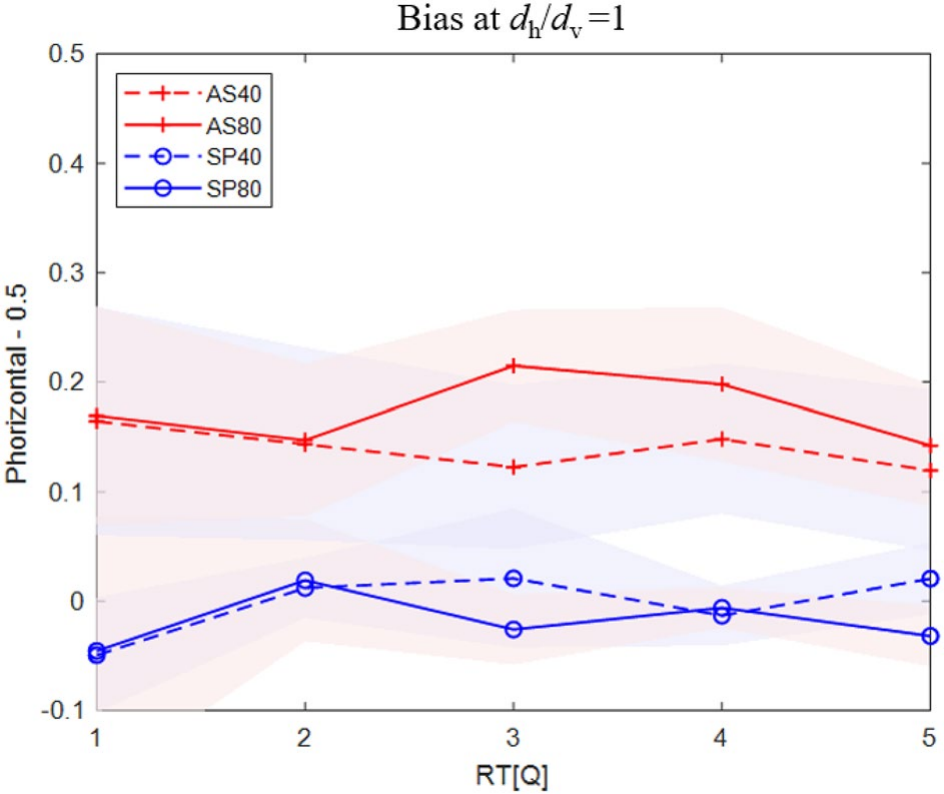
Perceptual bias as a function of reaction time (Q, quantile). SP40 and SP80 represent the spherical group with stimulus durations of 40 and 80 ms. AS40 and AS80 represent the astigmatic group with stimulus durations of 40 and 80 ms. The horizontal axes represent the different RT quantiles. Q1 is the fastest reaction time, and Q5 is the slowest. The vertical axes represent the bias, as an increase in horizontal reports relative to chance for stimuli with *d*_h_ = *d*_v_. Shaded areas show 1SEM.

Next, we investigated whether the bias found is due to a change in the decision strategy (response bias, criterion shift), or due to an asymmetric sensitivity along the two directions (horizontal and vertical displacements). To decide between these two options, we took advantage of a recently described relationship between bias and reaction time^19^, according to which decision bias is rapidly reduced in trials with increasing reaction times, whereas bias due to differential sensitivity is stable. Here we quantify bias as an increase in the probability of a horizontal response in ambiguous trials *d*_h_ = *d*_v_, depicted in Fig. 3 for five RT quantiles. The results clearly show a stable bias, independent of the reaction time. For the astigmatic group, the mean biases are 0.14 and 0.17 for the 40 and 80 ms conditions, respectively (both are statistically significantly positive, p < 0.01; Cohen’s *d* = 1.42). For the control spherical group, the mean biases are − 0.002 and − 0.02 for the 40 and 80 ms conditions, respectively, practically zero. There is no observable dependency of bias on the reaction time: the slopes of P_horizontal_ (Q) are − 0.08, − 0.0003, 0.01, and 0.0002 for the astigmatic 40 ms, astigmatic 80 ms, spherical 40 ms, and spherical 80 ms groups, respectively.

### Contrast sensitivity

**Figure 4:**
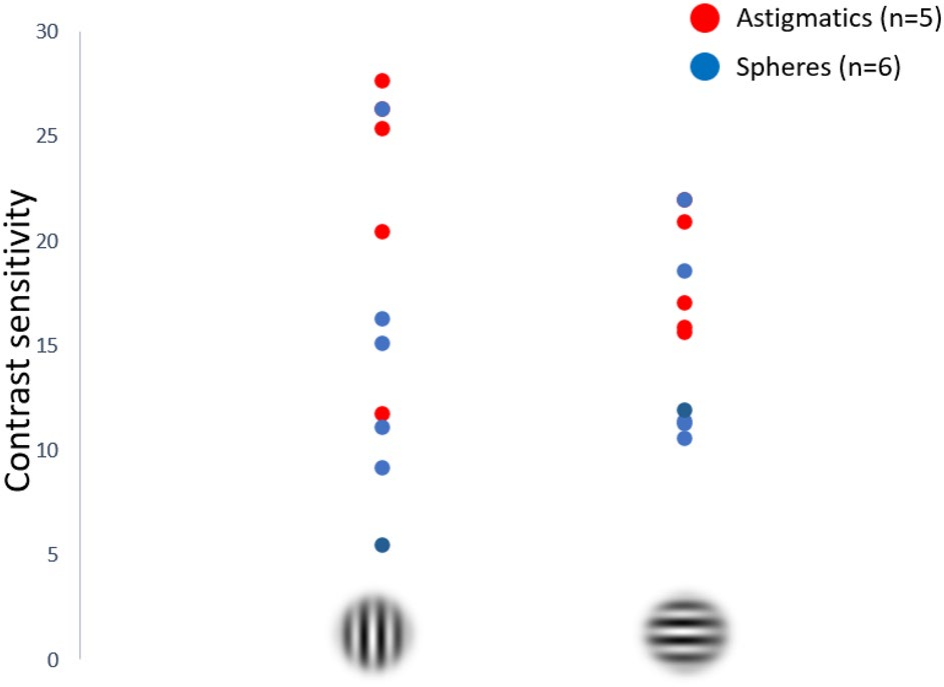
Contrast sensitivity for both groups studied according to horizontal and vertical orientation.

By examining the sensitivity along the meridians of astigmatism, we aimed to determine whether the orientation of astigmatism influenced the sensitivity and thus may explain the bias. Figure 4 shows the contrast sensitivity distribution between astigmatic and spheric individuals. The results show a somewhat better sensitivity in the vertical direction, though not statistically significant, respectively, for astigmatic (p = 0.21) and spherical participants (p = 0.99) and with no statistical difference between groups (p = 0.41). The results suggest that the bias observed in astigmatic participants is not due to a contrast sensitivity asymmetry of between 90 and 180 degrees. We did not observe a correlation between the contrast sensitivity of the horizontal and vertical ratio and the individual bias of each participant (r = − 0.082).

## Discussion

Our results show a clear perceptual bias for the astigmatic group and none for the spherical group. To rule out the effects of contrast sensitivity, we tested contrast sensitivity with vertical and horizontal grating patterns (Fig. 4). The results show for most participants (controls and astigmatic individuals), as expected^20^, a higher sensitivity for the vertical meridian, but no correlation with the perceptual bias in the grouping task. Thus, the measured bias implies that the astigmatic individuals perceive the horizontal dimension compressed relative to the vertical dimension, thus perceiving a horizontal organization at the array’s equilibrium point. Studies show that maturation of neural connectivity is experience dependent^21^ and that adaptation to uncorrected visual input during development may be inferred from the visual functions in adults^22^. We suggest that the horizontal compression results from a neuronal process developed to compensate for optical distortions during the early sensitive (so-called “critical’) period of visual development. Since the visual cortex matures before the optics settle, the compensation is permanent.

To exclude the possible effects of overcorrection, an optical adaptation that could vary by ± 0.25 D, another astigmatic participant ran the experiment after a period of adaptation to the overcorrected prescription. The results were similar; the bias was still persistent, suggesting that the bias is not the result of an overcorrection. We did not test participants with spherical correction because our previous experience^18^ indicated that participants with induced astigmatic blur show immediate bias that disappears after a few hours and days of adaptation. Thus, we expect that the newly induced astigmatic blur will show an immediate bias, which is not the issue that we wanted to test. Thus, giving correction on spherical participants is expected to produce a bias. Note that astigmatic participants are already beyond the process of adaptation; thus, the measured bias is a true representation of the neural adjustment to the blur effect.

Accommodation could play a role in the results. In our case, the pattern is robust enough to require an accommodative effort. Astigmatic accommodation usually has a small amplitude (< 0.25 D) under monocular viewing conditions and is present only in some eyes^23–25^; as mentioned above, we did not find any effect of ± 0.25 D of over-correction on the bias. Here, we can suggest that accommodation does not affect the bias obtained. We also used a pinhole lens of 1.8 mm on one of our astigmatic participants, monocular and binocular. The results were similar; the horizontal bias was in the same direction and equal to the fully optical correction and without the pinhole.

Our results show a much slower reaction time for the astigmatic group, compared with the spherical group, further supporting a non-typical developmental path for this group. We also observed a significant difference between the stimulus duration of 80 ms and 40 ms in both study groups, especially in the astigmatic group. The responses of both study groups were much faster with stimulus durations of 40 ms relative to 80 ms. These results can be explained by the reduced signal-to-noise ratio in the perceptual system with the 80 ms duration due to temporal integration, as indicated by the steeper slopes of the corresponding psychometric functions (see above, Fig. 2). This leads to a higher accuracy perception with increased uncertainty in ambiguous conditions where *d*_h_/*d*_v_ ~ 1. RT is known to increase with decision uncertainty^26^. Thus, the non-specific stimulus (ambiguous) may increase the processing demand, which results in increased RT.

We showed that the perceptual bias is not affected by the decision time. This result is consistent with a specific parametrization of the Drift Diffusion Model (DDM), used to predict the reaction time in decision tasks^27^. In DDM, decisions are modelled as a process of accumulating evidence, where a decision is made upon reaching a predefined likelihood threshold. Bias may result from prior information, providing evidence in favor of one response type over the other (criterion, decision bias, applied at the start of evidence accumulation as a prior), or from differences in the momentarily accumulated evidence between the two possible stimuli (sensitivity, one direction is more reliable than the other)^19^. Dekel and Sagi^19^ showed that the former predicts a fast-reducing bias with increasing reaction time, whereas the latter predicts a reaction time independent bias. The analysis explains a variety of perceptual biases as originating from changes in decision making or in sensitivity. Here we show that the observed bias is reaction time independent; thus, we suggest that the bias results from anisotropic *spatial* sensitivity. Note that our data were obtained from a small number of participants; therefore, it may limit the generalizability of our study to a population of astigmatic participants.

The bias found may represent a persisting adaptive correction, developed to compensate for the biased visual input during early life before the optical correction was applied, and only partly eliminated after its correction. In the context of the drift diffusion model, we interpreted the RT independent bias as an adaptive change in spatial mapping during development. This points to cortical astigmatism due to uncorrected astigmatism during the sensitive period.

## Methods

Our study used a paradigm similar to that of Yehezkel et al.^18^ by using a matrix of white dots^28^ as a stimulus. Eleven participants were enrolled in the experiment. All were students at Bar-Ilan University (their ages ranged from 18 to 30 years) with normal or corrected-to-normal visual acuity and who were unaware of the purpose of the study. The study protocol was approved by the Internal Review Board (IRB) of Bar-Ilan University. Informed consent was obtained from all participants and/or their legal guardian(s). All methods were performed in accordance with the relevant guidelines and regulations.

Each participant completed a full optometric exam that includes visual acuity (Snellen and logMar charts (ETDRS), autorefraction, retinoscopy, subjective and binocular tests (Cover test), stereo vision (Random dot), Van-Graef, fusional reserve, the amplitude of accommodation, as well as negative and positive relative accommodation. As part of the clinical procedure of the optometric exam, the refractive power of each meridian (astigmatism) was determined and used in the experiment. Table 1 reports the subjective refraction of each participant after retinoscopy.

**Table 1:** Refractive states of the participants*.*

Subject	Right eye	Left eye
NA	− 2.5/− 1.00 × 5	− 2.00/− 1.00 × 180
RB	− 0.75/− 1.25 × 180	− 0.25/− 1.50 × 180
RT	− 2.00/− 0.75 × 180	− 2.00/− 0.75 × 5
YR	pl/− 1.00 × 180	pl/− 1.00 × 180
SH	pl/− 1.25 × 175	− 0.25/− 1.00 × 175
AD	− 1.5	− 1.75
AK	− 0.25	− 0.5
AV	pl	− 0.25
TL	− 0.25	pl
SR	pl	pl
ED	− 0.75	− 0.75

We were aware of the comfortability of the participants with the NVIDIA glasses. To ensure accurate and reliable results, it was important to address the potential challenges associated with optical distortion and glasses misalignment during the experiment. To mitigate these concerns, all participants used their own glasses rather than a trial frame; thus, they were able to perform the experiment with their visual correction. This approach minimizes any discomfort or difficulties that may arise from using unfamiliar glasses, allowing participants to perform at their best. Furthermore, we verified that the optical center of each lens aligned with the participant's pupils, thus ensuring proper alignment and that the visual axis and the corrective power of the lenses are appropriately matched, thus, minimizing any potential image displacements or distortions that could affect the experimental measurements. By taking these precautions, the experiment aimed to eliminate potential confounding factors related to glasses misalignment or the presence of prism effects. This approach enhances the validity and reliability of the results by reducing unnecessary optical artifacts or biases associated with incorrect glasses usage.

A PC controlled the experiments, and the stimuli were displayed as a gray-level modulation on a BENQ XL 2411 color monitor (24″). The mean display luminance was 20 cd/m^2^, in a dark environment. The screen resolution was 1920X1080 pixels subtended a visual angle of 29° × 17°; gamma correction was applied.

We used 3D vision wireless stereoscopic polarized goggles (NVIDIA 3D stereoscopic glasses), which provide direct synchronization with the stimuli; thus, the participants are unaware of the stimulated eye.

Stimuli were viewed from 100 cm. A white dot matrix was presented on a gray background (Fig. 1A); each dot diameter was 6 pixels (6.4 min of arc), and the dot intensity was 45 cd/m^2^. The distance between the dots varied over the vertical or horizontal direction; the seven ratios (*d*_h_/*d*_v_) tested were 1.15, 1.1.10, 1.05, 1, 0.95, 0.90, and 0.85 (the higher ratio is usually perceived as columns).

Under most conditions, the margins of the matrix appeared rectangular due to the unequal spacing between the rows and columns, which may have interfered with the participants' judgment. To avoid such interference, the screen was covered with a round window with a radius of 5 cm so that the global form of the stimulus was circular across all experiments, with a diameter of 5.7 degree of the visual field.

The task consisted of reporting the perceptual organization of the display as horizontal (rows) or vertical (columns), with no feedback. There were 21 conditions: 7 *d*_h_/*d*_v_ values × 3 eye conditions (left, right, binocular). Each participant was tested with 40 trials on each condition (randomly mixed trials), totalling 840 trials per participant. Each trial was preceded by a fixation mark at the center of the display until the participants signalled her/his readiness using the computer mouse. Then a stimulus was presented for 40 or 80 ms, with the different durations presented in different blocks of trials. The participant responded by pressing the mouse buttons: right for vertical grouping and left for horizontal grouping.

**Figure 5:**
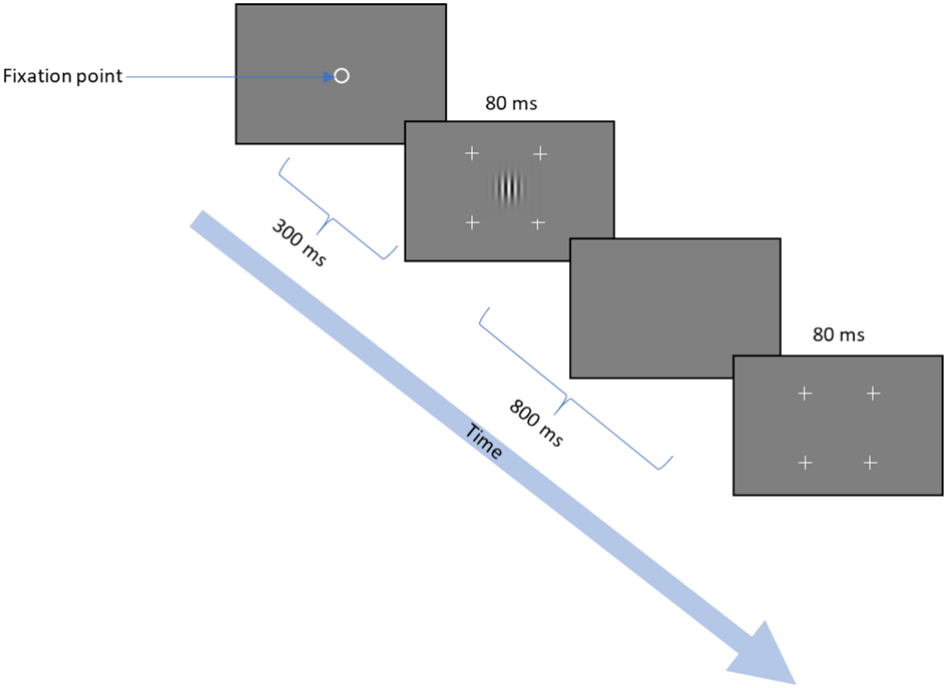
The contrast sensitivity paradigm. A Gabor target was presented at the center of the screen in one of two stimulation frames marked by 4 ‘+’s (2AFC). Participants contrast thresholds were estimated using an adaptive method.

To check for a possible anisotropy in contrast sensitivity due to astigmatism, we measured the oriented contrast sensitivity in the direction of the meridians of astigmatism. Stimuli were localized grey-level gratings (GP: Gabor Patterns, Fig. 5) with a spatial frequency of 12.8 cycles per degree (cpd) with equal wavelength and standard deviation (σ = λ = 0.078°). The GPs were added with a background luminance of 40 cd/m^2^. This spatial frequency was chosen because it represents a relatively high frequency that can challenge visual perception and potentially reveal any differences in sensitivities related to the tested astigmatism orientations.

To estimate the perceptual bias and the sensitivity to *d*_h_/*d*_v_, the following equation was fitted to the percent horizontal report as a function of *d*_h_/*d*_v_.

1 $${\varvec{P}}\left(x\right)=fe/2+ (1-fe) *(1-erf((x-1-\beta )/\sigma ))/2$$

where *x* = *d*_h_/*d*_v_, **β** is the bias, **σ** represent the width of the fitted function, and *fe* represents the finger errors (the fraction of unattended trials in which participants are guessing, assumed to be unbiased). All fits were with r^2^ > 0.98. Equation (1) was fitted to each participant’s empirical psychometric function.

The reaction time results, as a function of *d*_h_/*d*_v_, were fitted using the following equation.

2 $$RT\left(x\right)=RT0+RTd*exp(-0.5*(( x-1-\beta )/\sigma )^2)$$

where **β** represents the bias, *x* = (*d*_h_/*d*_v_), **σ** represents the sensitivity of RT to *d*_h_/*d*_v_. *RT0* is the base RT, representing all stimulus-independent perceptual and motor processes, and *RTd* represents the added stimulus-dependent decision time at maximal uncertainty.

We used a paired two-tailed t-test and 2-way ANOVA to compare the bias and reaction time between astigmatic and spherical groups in two the different stimulus time-duration conditions within the same participants. In addition, Cohen’s *d* effect size was also calculated.

## Data Availability

The datasets used and/or analysed during the current study available from the corresponding author on reasonable request.
